# Development of an augmented virtuality framework using user-defined passthrough surfaces

**DOI:** 10.1016/j.mex.2026.104017

**Published:** 2026-06-26

**Authors:** Abdul Hannan Bin Zulkarnain, Attila Gere

**Affiliations:** Department of Postharvest, Supply Chain, Commerce and Sensory Science, Institute of Food Science and Technology, Hungarian University of Agriculture and Life Sciences, Villányi út 29-31, Budapest, H-1118, Hungary

**Keywords:** Virtual reality, Mixed reality, Passthrough, Unity, OpenXR

## Abstract

An augmented virtuality framework is presented in which the physical scene is admitted only through developer-defined geometric apertures, while all other pixels are rendered virtually. The scene initialises as a black field and uses surface-projected, overlay-style compositing so that the camera stream is visible only on circular or square meshes registered as projection surfaces. Tracking origin is configured to minimise unintended recentring, and the same method can operate either as a minimal black scene or within an optional three-dimensional environment.

Main features of the framework are as follows:

deterministic compositing that binds real imagery to user-defined meshes and prevents leakage outside apertures.

stable alignment between virtual geometry and the physical workspace during head motion achieved through standard XR configuration.

a flexible scene recipe that supports circular or square apertures and optional contextual environments without altering projection logic.

The framework is intended for sensory and consumer studies that require real product interaction under controlled context and generalises to training, human factors, and rehabilitation scenarios requiring constrained visibility of the real world.

## Graphical abstract

**Figure:**
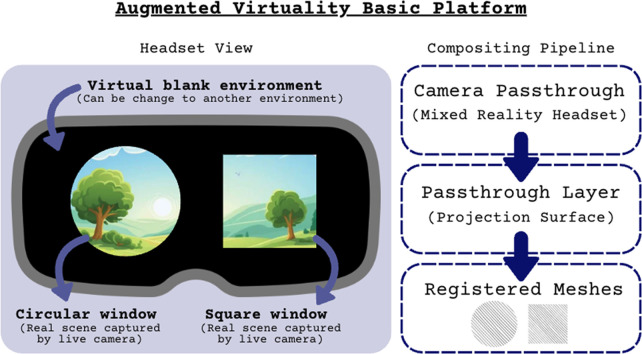

**Specifications table**

**Table:** 

**Subject area**	Food Science
**More specific subject area**	*Immersive Technology for Sensory Testing*
**Name of your method**	*Surface-Projected Augmented Virtuality with Circular or Square Passthrough Windows*
**Name and reference of original method**	*N/A*
**Resource availability**	*Suggested device: Meta Quest 3* *s (tested). Compatible with Meta Quest Pro and 3 (full-color passthrough) and Meta Quest 2 (grayscale passthrough).* *Software needed: Unity 6000.1.12f1.* *Packages required (Unity Package Manager):* *– XR Plugin Management (enable OpenXR for Android)* *– OpenXR Plugin (com.unity.xr.openxr)* *– Meta XR SDK — All-in-One (com.meta.xr.sdk.allinone)* *Packages optional (for interaction/testing):* *– XR Interaction Toolkit (com.unity.xr.interaction.toolkit)* *– XR Hands (com.unity.xr.hands)* *– Universal Render Pipeline (URP)* *Build target / settings: Android, ARM64, Vulkan,* Min *API 29+.* *Software repository:*https://github.com/matedigitalsensorylab/Raw_AV *Developer documentation/manual:*https://github.com/matedigitalsensorylab/Raw_AV/blob/main/README.md *License: MIT (in repository).*

## Background

Sensory and consumer evaluation needs both experimental control and ecological validity [1]. Highly controlled testing rooms reduce unwanted variation, but they can remove the cues that matter in everyday decisions, such as the look of the real product on a real table [2]. Fully virtual scenes give strong control but often replace real foods with digital models, and this can change how people combine sight, touch, smell, and taste [3]. Camera passthrough shows the real world directly, but it can also show distracting backgrounds and uncontrolled lighting [4].

Augmented virtuality (AV) offers a balanced solution. In this approach, the virtual scene remains in charge, and only chosen parts of the real world are shown [5]. The framework here does that by projecting the live camera image onto specific meshes that act like windows. Everything outside the windows is drawn by the graphics engine and can be standardized. The simplest version is a black scene that removes all background context. A richer version uses a simple virtual room that offers orientation without introducing strong visual cues.

**Fig. 1:**
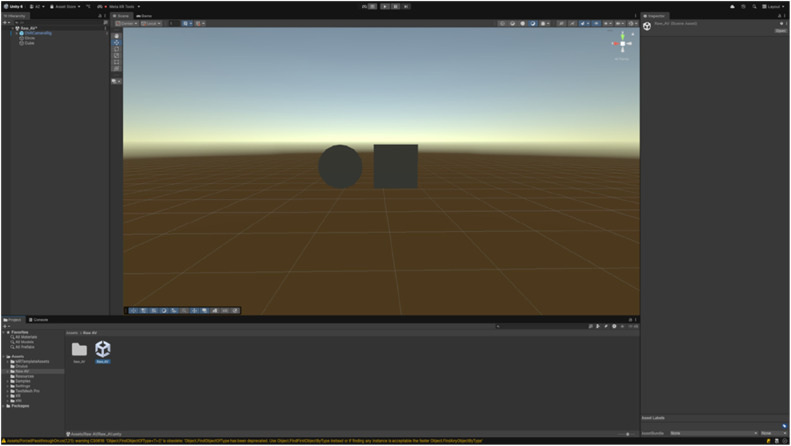
Scene overview showing OVRCameraRig, the OVR Passthrough Layer, and the circular and square meshes used as user-defined projection surfaces.

This method follows the concept introduced in the related research article by Zulkarnain et al. [5]. That article explains why augmented virtuality can improve ecological validity in consumer studies. The present paper provides a clear implementation that researchers can use as a starting point for their own experiments. The central idea is straightforward: the compositor maps the camera image onto developer-selected meshes and draws it only on those surfaces, above the rest of the virtual scene. Circular and square windows are created from simple primitives, making their size and placement easy to report and reproduce. Because these are ordinary scene objects, researchers can vary window number, size, and spacing as experimental factors without changing the compositing logic (Fig. 1).

In contrast to full scene passthrough setups that render the camera feed across the entire field of view, the present implementation constrains real-world imagery to explicitly defined apertures. Accordingly, the contribution of the present paper is a reproducible implementation recipe for aperture-constrained augmented virtuality, rather than a depth-aware occlusion system. Unlike depth-aware mixed-reality approaches that use depth or scene reconstruction to support occlusion, this framework treats passthrough visibility as a deterministic mask defined by developer-registered projection meshes. This prioritises controlled, reportable exposure of real-world imagery and reproducible experimental conditions over physically realistic depth-based blending.

The present framework relates to mixed-reality techniques that selectively display real-world imagery. Modern VR SDKs support surface-projected passthrough or selective passthrough, where the headset projects camera video onto specified virtual surfaces rather than relying on automatic environment depth reconstruction [6]. In the present approach, user-defined meshes act as opaque windows into reality. This can be understood as a constrained form of computer-mediated reality because real-world video is composited only on predefined geometry without scene reconstruction [7]. Unlike depth-aware mixed reality, the real-world image is overlaid at full opacity on the registered meshes, so virtual objects do not occlude real objects inside the apertures. This supports reproducible and deterministic apertures, but it does not provide physical occlusion, object-aware compositing, or scene reconstruction. The framework is therefore positioned as a constrained video-see-through augmented virtuality technique rather than a depth-aware mixed-reality system.

## Method details

**Fig. 2:**
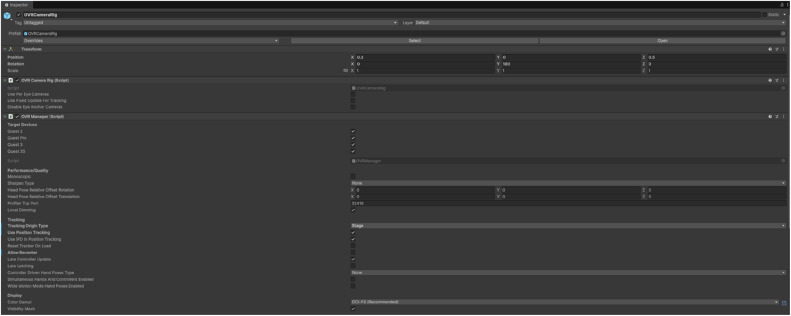
Unity Inspector view of the OVRCameraRig object showing the OVRManager component settings used in the present framework to support stable tracking during ordinary runtime use. This figure shows the practical tracking configuration underlying the spatial behaviour of the augmented virtuality system, including the selected tracking origin, enabled position tracking, and recentre-related options intended to reduce unintended spatial shifts of the user-defined passthrough apertures. Because the circular and square projection surfaces are registered in the same tracked coordinate frame, stable headset-to-scene alignment is important for maintaining the apparent position of the apertures during normal operation.

This implementation is specified for Unity 6000.1.12f1 (Unity Technologies, Unity Software Inc., San Francisco, California, US) with the Universal Render Pipeline (URP) and targets Android using IL2CPP, ARM64, and Vulkan. URP Global Settings and a URP Renderer are assigned in Project Settings → Graphics and mirrored in Quality so all tiers resolve to the same pipeline. To create a neutral visual baseline, the skybox is removed in Lighting and ambient light is set to a constant black. A single render camera (the CenterEyeAnchor under OVRCameraRig) is used and configured in URP with Background = Solid Colour (black) so pixels not filled by mesh rendering or camera composition remain black (Fig. 1). Tracking is configured to avoid automatic recentring during boundary prompts; this practical choice keeps the user-defined windows visually stable in the headset and prevents sudden origin jumps (Fig. 2). Because the projection surfaces are fixed in the tracked coordinate frame, the framework depends on stable coordinate-space behaviour between the headset tracking origin, the registered projection-surface meshes, and the physical workspace. Runtime reset, user-initiated recentring, boundary reset, environmental relocalisation, or visible cumulative drift may shift this coordinate frame and reduce aperture alignment quality. In practical deployment, aperture transforms should be checked after any recentring event, boundary reset, visible drift, or prolonged session, and recalibrated before data collection continues if alignment has changed. Software versioning and setup instructions are provided in the public repository and README listed in the Specifications table.

**Fig. 3:**
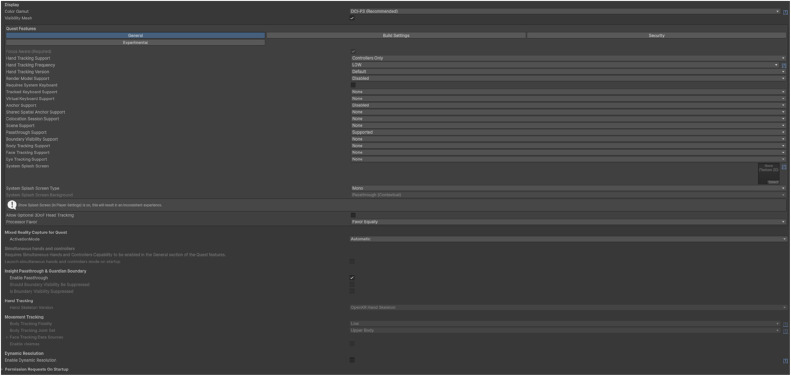
Unity Project Settings view showing the Android OpenXR configuration together with the Meta XR runtime features required for the present implementation. This figure shows the platform-level functions needed for headset tracking, controller or hand input, and camera passthrough support, which together allow the application to render live real-world imagery onto developer-defined projection surfaces at runtime. These settings establish the runtime prerequisites for the constrained augmented virtuality framework before scene-level compositor parameters are applied.

Runtime support is then enabled in Project Settings → XR Plug-in Management by turning on OpenXR for Android and enabling the Meta features required for tracked input and camera passthrough. This ensures the application receives headset poses, controller or hand input, and access to the passthrough feed at runtime (Fig. 3). For readers who want the shortest reproducible setup path, the implementation can be reduced to five steps: (1) add OVRCameraRig and remove other active cameras; (2) enable OpenXR for Android and the required Meta passthrough features; (3) add one OVR Passthrough Layer and set Projection Surface = User Defined, Placement = Overlay, and Opacity = 1.0; (4) create flattened cylinder or cube meshes at the intended physical workspace; and (5) register those meshes in the Projection Surfaces list or by AddSurfaceGeometry at startup.

**Fig. 4:**
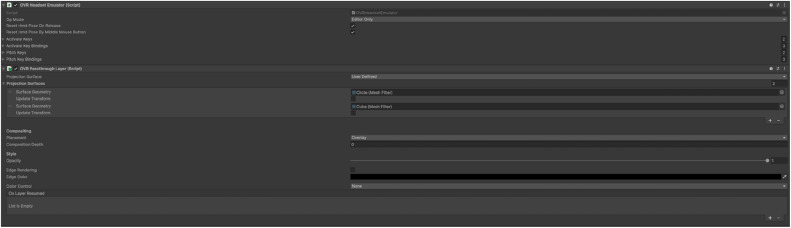
Unity Inspector view of the OVRPassthroughLayer component showing the compositor configuration used to constrain the live camera feed to developer-registered meshes. The figure shows the key parameters of the method: Projection Surface = User Defined, which restricts passthrough rendering to explicitly assigned surfaces rather than the full field of view; Placement = Overlay, which composites the camera image above virtual scene content without depth-based occlusion; Texture Opacity = 1.0, which preserves full passthrough visibility inside the apertures; and the Projection Surfaces list, where the circular and square meshes are registered. Together, these settings implement the deterministic aperture-based masking logic described in the manuscript.

Composition of the live camera feed uses an OVRPassthroughLayer configured with Projection Surface = User Defined, Placement = Overlay, and Opacity = 1.0. This configuration projects the live camera image only onto the registered circular or square meshes and composites it above the virtual scene without depth-based occlusion. The black baseline is produced by the scene camera background, while the passthrough feed remains visible only inside the assigned projection surfaces. This keeps masking deterministic. Aperture visibility is controlled by the registered mesh silhouettes, not by scene depth, object recognition, or spatial reconstruction. The method therefore supports controlled aperture-based exposure rather than physically realistic depth-aware mixed reality (Fig. 4).

**Fig. 5:**
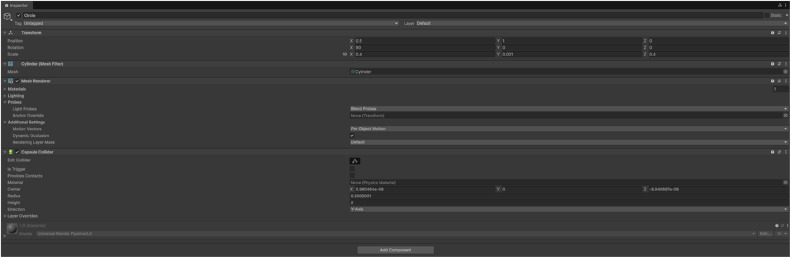
Unity inspector view of the circular projection surface created from a standard cylinder primitive flattened along its Y axis to form a disc-like aperture. The figure shows the transform, mesh, renderer, collider, and material settings associated with this circular passthrough window. In the present framework, this object functions as one of the registered projection surfaces onto which the live camera image is composited. Its position, rotation, and scale therefore determine the location, orientation, and apparent size of the visible circular real-world aperture in the headset view.

**Fig. 6:**
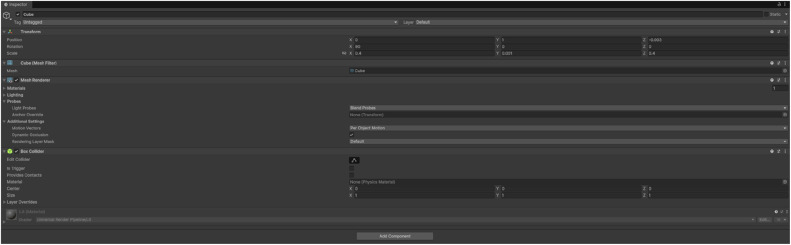
Unity Inspector view of the square projection surface created from a standard cube primitive flattened along its Y axis to form a planar square aperture. The figure shows the transform, mesh, renderer, collider, and material settings associated with this square passthrough window. As with the circular window, this mesh is registered with the passthrough compositor and functions as a geometric mask that defines where the live camera image is visible. Its transform parameters therefore determine the spatial placement and apparent dimensions of the square real-world viewing region in the final headset display.

Windows are authored from standard primitives so their geometry and transforms can be reported and reproduced precisely. A Cylinder flattened along its Y axis produces a circular window; a Cube flattened along its Y axis produces a square window. Typical visible diameters or side lengths are 0.25–0.35 m with a thickness of about 0.01 m. Each window is positioned and oriented with ordinary transform values so that it appears at the intended location in front of the user. Because these are standard meshes, their poses (position, rotation, scale) can be logged, versioned, and replicated across studies (Fig. 5, Fig. 6).

Meshes are registered with the compositor using either of two equivalent approaches. In the inspector approach, the Projection Surfaces list on the OVR Passthrough Layer is sized to the number of windows and each window’s Mesh Filter is assigned manually. In the programmatic approach, the application locates the layer at startup and calls AddSurfaceGeometry for each window. If a mesh is intended to function only as a projection mask, its MeshRenderer can then be disabled so the geometry does not remain visibly rendered in the scene. A minimal example is provided below and mirrored in the public repository.

using UnityEngine;

public class PlateRegister : MonoBehaviour

{

public OVRPassthroughLayer passthrough;

public MeshRenderer [] plates;

void Start()

{

if (passthrough == null)

passthrough = FindObjectOfType<OVRPassthroughLayer>();

foreach (var plate in plates)

{

if (plate == null || passthrough == null) continue;

passthrough.AddSurfaceGeometry(plate.gameObject, false);

// Optional: disable visible rendering if the mesh should act only as a mask

plate.enabled = false;

}

}

}

The same logic is implemented in the public repository scripts PlateRegister.cs, EnableOVRPassthrough.cs, and ForcedPassthroughOn.cs, which are named here explicitly to make the reproducibility path easier to follow.

When additional spatial cues are beneficial, a minimal 3D environment can be enabled under an EnvironmentRoot. Toggling this environment does not change masking logic: with the environment disabled, regions outside the apertures remain black; with it enabled, those regions display the virtual room while passthrough remains confined to the registered surfaces. For building, the scene is added to Scenes In Build and compiled for Android with IL2CPP, ARM64, Vulkan, targeting the headset’s refresh rate. High dynamic range (HDR) is kept off for headroom, and Multisample Anti-Aliasing (MSAA) is set to 2× or 4× depending on aliasing tolerance. Real-time shadows are avoided for the black-field condition and used sparingly when the environment is enabled [8]. Textures are imported with Adaptive Scalable Texture Compression (ASTC) compression. On first launch, the runtime requests camera permission and activates passthrough according to project configuration.

Optional interaction can be added via the Meta Interaction SDK without altering the augmented-virtuality logic. Controller prefabs with ray and grab interactors enable pointing and manipulation; interactable objects use a Collider and the appropriate Grab Interactable component. Hand interaction uses hand prefabs with hand-grab, poke, and ray interactors. Unity UI canvases are driven by the OVR Input Module when Meta rays are present. Because passthrough visibility is bound only to the registered projection surfaces, these interaction features do not alter the masking constraint.

For hardware, development and verification were performed on Meta Quest 3S. The same configuration runs unmodified on Meta Quest 3 and Pro. On Meta Quest 2, the compositing behaviour is the same, but the device provides grayscale passthrough, so real-world imagery inside the windows appears in grayscale. No changes to project settings or scripts are required to support these devices. Although the masking logic is unchanged across supported devices, differences in passthrough sensor quality, grayscale versus colour rendering, noise, and edge sharpness may influence how clearly aperture boundaries are perceived and how visually continuous the mixed scene appears.

For reproducibility, the essential conditions are: Unity 6000.1.12f1 with URP assigned in Graphics and Quality; a single OVRCameraRig with one active render camera (CenterEyeAnchor) using a black solid background; one OVR Passthrough Layer set to User Defined and Overlay; registered circular or square window meshes with documented transforms; and, when using the programmatic path, disabled MeshRenderers on the windows so the geometry functions solely as a projection mask.

## Methods validation

**Fig. 7:**
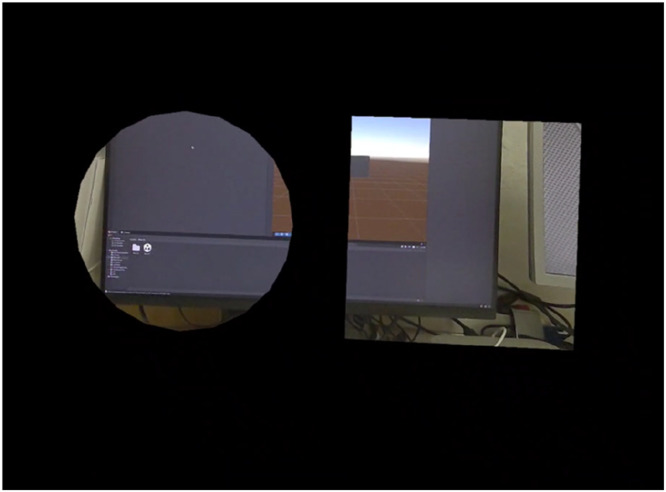
Headset view demonstrating deterministic masking: the live camera image appears only inside the circular and square apertures, while the remainder of the field resolves to the black baseline.

**Fig. 8:**
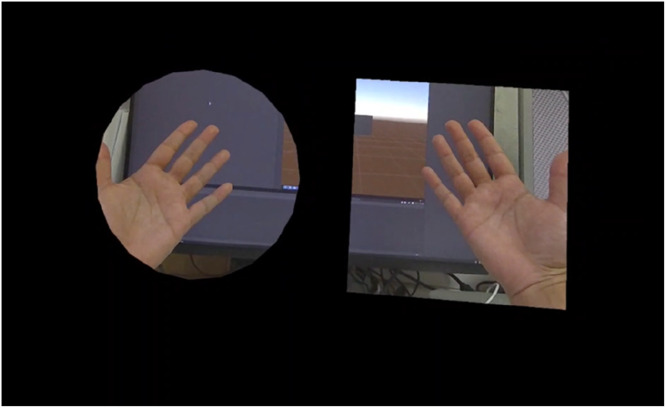
Same scene after a small head movement. The apertures remain fixed in the virtual frame while the real view shifts inside them with normal motion parallax.

The validation presented here is qualitative and image-based. It demonstrates deterministic masking, draw order, and stable apparent behaviour under typical head motion, but it does not constitute a quantitative performance evaluation. No controlled user trial, geometric deviation measurement, latency measurement, frame-timing analysis, or perceptual rating study was performed. The examples should therefore be interpreted as implementation checks under tested conditions rather than evidence of general system accuracy or perceptual performance. Two headset point-of-view images (Fig. 7, Fig. 8) were captured with the configuration described above: a single render camera with a black background, an OVRPassthroughLayer set to User Defined projection surfaces and Overlay placement, and two registered projection meshes consisting of a flattened cylinder and a flattened cube.

Fig. 7 demonstrates strict masking. The passthrough image is visible only within the circular and square apertures, while the remainder of the field resolves to the black baseline. The boundaries follow the registered mesh geometry and show no visible leakage outside the defined apertures.

Fig. 8 shows the same setup after a small head movement. The projection surfaces remain fixed relative to the virtual scene, while the real view shifts inside each aperture with normal motion parallax, consistent with projecting the camera stream onto fixed meshes in the tracked coordinate frame. Because overlay placement is used, passthrough is composited above virtual content inside the apertures and does not participate in depth-based occlusion. Under real interaction conditions, partial crossing of an aperture boundary by the user’s hands or by manipulated physical objects may produce a visible cut line or edge discontinuity, which is an expected consequence of overlay-based masking rather than depth-aware occlusion.

No instrument-based benchmarking of geometric deviation or system latency was performed for this initial methods paper, and the validation does not claim quantitative error bounds. Tracking stability was assessed under normal head motion during runtime and was not separately stress-tested under boundary reset prompts or user-initiated recentre events. Where quantitative alignment accuracy, edge-artefact characterisation, or timing measurements are required, these can be added in application-specific studies without changing the core masking method described here. Taken together, these observations support the core implementation claim of the method: passthrough remains confined to developer-defined apertures and preserves stable apparent registration during ordinary runtime use under the tested conditions, while robustness under recentring events, longer sessions, and quantitative benchmarking is reserved for future application-specific studies.

### Limitations

This method reveals real objects only through developer-defined apertures in an otherwise virtual scene. Accurate perceptual alignment therefore depends on each projection-surface mesh matching its intended physical counterpart. Because the system depends on the headset’s built-in tracking, tracking error, cumulative drift, runtime reset, boundary reset, or user-initiated recentring may shift the coordinate frame and reduce aperture alignment quality. A recalibration or transform check is therefore advisable after any tracking reset, recentring event, visible drift, or extended session.

The passthrough layer is composited as an overlay to ensure deterministic masking and consistent draw order. This improves predictability for controlled conditions, but it does not support physically plausible occlusion of the real view by virtual objects within the apertures. Because the implementation uses an overlay mask, any real object that crosses an aperture edge will be sharply “cut off.” For example, a hand moving through the window will show a visible seam at the boundary (see Fig. 8). These artefacts tend to be more noticeable in high-contrast scenes or on devices with lower-resolution passthrough cameras, where the sharp transition is easier to see.

Future work could include optional quantitative benchmarking workflows to characterise system performance under application-specific conditions. These may include aperture alignment deviation, edge visibility under different contrast conditions, and frame-timing profiles. Such measurements would complement the qualitative implementation checks presented here without changing the core masking method. Cross-device guidance and optional interaction modules (e.g., eye tracking for fixation checks and hand tracking for standardised handling cues) will be developed where supported, in line with broader VR applications in food science [9].

## Applications and extensibility

The framework is intended as a base for augmented virtuality in sensory analysis, preserving real product handling while standardising the surrounding visual context through constrained passthrough apertures. It can also support training and skills assessment by revealing only a real tool and work surface, human factors studies that vary peripheral visibility in a controlled way, and rehabilitation tasks that gradually change window number and size. Using the same idea on other headsets is practical as long as the device provides an interface to project the camera image onto developer chosen meshes and to draw that image on top of the scene.

## Ethics statements

No human or animal subjects were involved in the development and technical validation of the method. Any application in human studies requires prior ethics approval and informed consent in accordance with institutional policy.

## CRediT authorship contribution statement

**Abdul Hannan Bin Zulkarnain:** Conceptualization, Methodology, Software, Validation, Visualization, Writing – original draft, Writing – review & editing. **Attila Gere:** Conceptualization, Methodology, Validation, Visualization, Writing – original draft, Writing – review & editing.

## Declaration of competing interest

The authors declare that they have no known competing financial interests or personal relationships that could have appeared to influence the work reported in this paper.

## Data Availability

I have shared the link to my data and can be found at "Resource availability" part

## Appendix Supplementary materials

Image, video 1 dummy alt text
